# Biomarkers of cereal food intake

**DOI:** 10.1186/s12263-019-0651-9

**Published:** 2019-10-12

**Authors:** Rikard Landberg, Kati Hanhineva, Kieran Tuohy, Mar Garcia-Aloy, Izabela Biskup, Rafael Llorach, Xiaofei Yin, Lorraine Brennan, Marjukka Kolehmainen

**Affiliations:** 1 Department of Biology and Biological Engineering, Division of Food and Nutrition Science, Chalmers University of Technology, SE-412 96 Gothenburg, Sweden; 2 Institute of Public Health and Clinical Nutrition, University of Eastern Finland, P.O. Box 1627, FI-70211 Kuopio, Finland; 3 Nutrition and Nutrigenomics Unit, Department of Food Quality and Nutrition, Research and Innovation Centre, Fondazione Edmund Mach, San Michele all’Adige, 38010 Trento, Italy; 4 Biomarkers and Nutrimetabolomic Laboratory, Department of Nutrition, Food Sciences and Gastronomy, Food Technology Reference Net (XaRTA), Nutrition and Food Safety Research Institute (INSA-UB), Faculty of Pharmacy and Food Sciences, Campus Torribera, University of Barcelona, Barcelona, Spain; 5 CIBER de Fragilidad y Envejecimiento Saludable (CIBERFES), Instituto de Salud Carlos III, Barcelona, Spain; 6 UCD School of Agriculture and Food Science, Institute of Food and Health, Belfield, Dublin 4, Ireland

**Keywords:** Cereals, Biomarkers, Whole grain, Alkylresorcinols, Cinnamic acids, Phenolic acids, Benzoxazinoids, Avenanthramides, Avenacosides

## Abstract

**Background/objectives:**

Cereal foods are major contributors to the daily energy, protein, and dietary fiber intake all over the world. The role of cereals in human health is dependent on whether they are consumed as refined or whole grain and on cereal species. To unravel the underlying mechanisms of health effects attributed to specific cereal foods and to provide more precise dietary advice, there is a need for improved dietary assessment of whole-grain intake. Dietary biomarkers of specific cereals, different fractions or cereal-containing foods could offer such a possibility. The aim of this review was to summarize the current status on biomarkers of different cereals, fractions, and specific cereal foods.

**Subjects and methods:**

A literature review was conducted and putative biomarkers of different cereals and pseudo-cereals (wheat, oats, rye, barley, rice, and quinoa) as well as for different grain fractions (whole grain, refined grain, bran) and foods were summarized and discussed.

**Results:**

Several putative biomarkers have been suggested for different cereals, due to their unique presence in these grains. Among the biomarkers, odd-numbered alkylresorcinols are the most well-studied and -evaluated biomarkers and reflect whole-grain wheat and rye intake. Even-numbered alkylresorcinols have been suggested to reflect quinoa intake. Recent studies have also highlighted the potential of avenanthramides and avenacosides as specific biomarkers of oat intake, and a set of biomarkers have been suggested to reflect rice bran intake. However, there are yet no specific biomarkers of refined grains. Most biomarker candidates remain to be evaluated in controlled interventions and free-living populations before applied as biomarkers of intake in food and health studies.

**Conclusion:**

Several putative biomarkers of different cereals have been suggested and should be validated in human studies using recently developed food intake biomarker validation criteria.

## Background

Cereal foods constitute a major food group, and they are one of the main contributors to energy and dietary fiber intake in the diet all over the world [1]. Today, cereals are mostly consumed as refined grains, i.e., the nutrient-rich bran and germ have been removed. However, whole grain-based foods, i.e., where all parts of the grain kernel are present in cracked, intact, or milled form, is reaching wider acceptance among consumers. Whole-grain foods are advocated by governmental authorities in many countries due to beneficial health effects [2]. Whole-grain food intake has been consistently associated with lower risk of non-communicable diseases such as obesity, cardiovascular disease, type 2 diabetes, and colorectal cancer in different populations [3–7], whereas a high intake of refined grains has been associated with no or even adverse health outcomes [4, 8, 9]. Whole grains are rich in dietary fiber, vitamins, minerals, unsaturated fatty acids, and phytochemicals, all of which may contribute to protective effects [10]. Moreover, the native structure of the food raw material as well as process induced structural changes that might encapsulate nutrients, slow digestion, and absorption could also play a role for health especially in the gut [11]. Specific dietary fibers, such as β-glucans, the fructans, and resistant starches, including process-induced resistant starch commonly found in whole grains, could induce gut microbiota fermentation in the large intestine, which has been linked to beneficial health effects [12, 13].

While observational studies [3–5, 14, 15] have provided consistent evidence for a beneficial effect of high whole-grain intake in chronic disease prevention, the outcomes from short- to long-term randomized controlled trials are less consistent [16, 17]. Yet, randomized controlled trials investigating the role of whole-grain intake for primary prevention of non-communicable disease have not been reported, due to large challenges related to costs of such trials and problems to ensure compliance over long periods of time. Instead, short-term dietary interventions to address effects on established biomarkers or risk markers for non-communicable diseases have been conducted to investigate the role of separate grains and mixed whole grains on cardiometabolic risk factors, but also short- to intermediate-term studies have been shown to have problems with compliance [17–19].

A problem in observational studies is that whole-grain intake is associated with an overall healthy lifestyle and dietary pattern, and it is difficult to study the impact of whole grains per se on health outcomes, despite adjustment for confounding factors [20]. Moreover, and probably more importantly, the dietary instruments typically used to assess whole-grain intake in observational studies lack the precision required to accurately measure the intake of different grains separately. Various cereals differ in the content and composition of constituents thought to exert health effects, but this has typically not been accounted for in observational studies [21–23]. Another challenge for accurate assessment of the habitual whole-grain intake with common self-reporting techniques such as food frequency questionnaires, dietary recalls, or food records is that consumers may have difficulties in distinguishing/identifying different grains and to understand portion sizes, in addition to well-known effects of under- and over-reporting. Furthermore, whole-grain products have a large variation in whole-grain content, which affects the precision of estimates [24]. Moreover, no uniform definition of whole-grain products or serving size has been used across studies [25–27]. This may lead to misclassification, which is likely to attenuate the association between whole grain and disease towards null and preventing existing associations with disease outcomes to be revealed or cause underestimation of associations that may be stronger than observed [28].

Using dietary biomarkers that reflect the intake of specific whole grains, grain fractions, and refined grains could be a strategy to improve whole-grain intake ranking in observational studies as well as to address compliance in dietary intervention studies [29–32]. Dietary biomarkers may also be combined with traditional methods to improve the accuracy of intake estimations [33]. However, only a few dietary biomarkers that reflect specific whole-grain intakes have been suggested [34] whereas no biomarkers of refined grains have been described.

The aim of the present review is to provide an updated overview of potential biomarkers of different cereals, including different species, whole grains, refined grains as well as specific grain fractions.

## Literature search

**Fig. 1:**
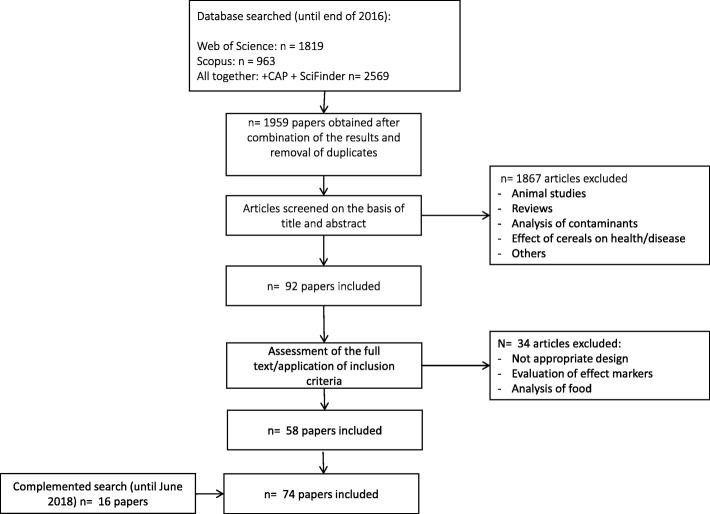
Flow diagram of study selection

The reviewing process conducted made use of all elements of the PRISMA statement [35] that were relevant for a search for literature on cereal biomarkers. In brief, original research papers and reviews were searched in at least two databases, such as CAB Abstracts, Scopus, and ISI Web of Knowledge using combinations of the grouped search terms (biomarker* OR marker* OR metabolite* OR biokinetics OR biotransformation OR metabolism) AND (trial OR experiment OR study OR intervention) AND (human* OR men OR women OR patient* OR volunteer* OR participant) AND (urine OR plasma OR serum OR blood OR excretion) AND (intake OR meal or diet OR ingestion OR consumption OR eating OR drink* OR administration) AND (wheat* OR rye OR oat* OR barley OR rice OR sorghum OR corn OR maize OR germ OR endosperm OR bran OR wholegrain OR whole-grain OR “whole grain” OR bread* OR cereal* OR flour* OR pasta*). The research was limited to papers in the English language, while no restriction was applied for the publication date. The research papers with identification or use of potential biomarkers of cereal intake were selected by one or more skilled researchers from the list of retrieved references in a process outlined in Fig. 1. Additional papers were identified from reference lists in these papers and from reviews or book chapters identified through the search. For each potential biomarker identified an additional search was conducted with (“the name and synonyms of the compound” OR “the name and synonyms of any parent compound”) AND (biomarker* OR marker* OR metabolite* OR biokinetics OR biotransformation) in order to identify potential other foods containing the biomarker or its precursor. In this second step, PubMed, Scifinder, and Google Scholar were also used as search platforms, along with the databases listed above. This second search was used to evaluate the apparent specificity of proposed biomarkers. The literature search was conducted in 2016 and papers published until the end of 2016 were included. A complementary search was conducted in a similar way and additional papers published until June 2018 were added to the literature list.

### Classification of cereal biomarkers

Dietary biomarkers may reflect intake or efficacy, depending on whether the biomarker is a compound resulting from the consumed dietary item, or if it is an endogenous metabolite reflecting the change in the host metabolic homeostasis evoked by the diet. A comprehensive classification system [36] and a validation scheme for biomarkers of food intake (BFI) has recently been developed by the FoodBall consortium [37]. In this classification, biomarkers were divided into six classes: food compound intake biomarkers (FCIBs), biomarkers of food or food component intake (BFI), dietary pattern biomarkers (DPBs), food compound status biomarkers (FCSBs), effect biomarkers, and physiological or health state biomarkers. According to the traditional classification of dietary biomarkers, FIBs can be classified as *recovery* and *concentration* biomarkers depending on their characteristic. In the FoodBall classification system, the recovery biomarkers are included in the health state biomarkers. Recovery biomarkers reflect the balance between intake and excretion of a specific chemical component on an absolute scale over a specific time period whereas concentration biomarkers are correlated with intake [38]. Recovery biomarkers represent the best standard and can be used to calibrate other dietary instruments [39]. Sometimes prediction biomarkers are mentioned as a third category, falling in between the recovery biomarkers and concentration biomarkers [40]. Most biomarkers belong to concentration biomarkers [41].

### Targeted and untargeted metabolomics approaches for discovery of cereal biomarkers

Following recent advances over the last years, metabolomics has become a fundamental tool to study changes in molecular phenotype caused by molecules inherent to different exposures, including diet, and their interaction with host risk of disease or other outcomes [42, 43]. Generally speaking, biomarkers may be discovered in different sample matrices such as plasma, erythrocytes, urine, adipose tissue, hair, and nail clippings. Each matrix has their challenges, but in general, dietary biomarkers are typically present at lower concentrations in plasma compared with urine, to which many biomarker molecules or metabolites thereof are excreted. Only a few biomarkers are accumulated in adipose tissue and can be detected in hair. For cereals, individual biomarkers that reflect specific cereal foods have been detected in plasma, urine, and adipose tissues and by utilizing chemometric, multivariate tools, there are new possibilities to use combinations of several biomarkers, i.e., biomarker panels, which may improve prediction of outcomes as well as monitoring of compliance or measuring food intake compared with single concentration biomarker [44].

Metabolomics methodologies can be divided into targeted and untargeted approaches [45]. In targeted metabolomics, a defined set of well-characterized and annotated metabolites are analyzed typically in quantitative platforms such as triple quadrupole mass spectrometry (QQQ-MS) utilizing pure chemicals as standards. Targeted metabolite analyses have been used to analyze compounds known or suspected to be putative biomarkers of specific foods, such as odd-numbered alkylresorcinols (whole-grain wheat and rye) [46], even-numbered alkylresorcinols (quinoa) [47], avenanthramides and avenacosides (oats) [48, 49]. In contrast, untargeted approaches aim at maximizing the metabolite coverage in a set of biological samples, even though the vast majority of measured metabolic features remain unidentified. A common analytical platform for profiling assays is quadrupole time-of-flight mass spectrometry (QTOF-MS) hyphenated with chromatographic separation in either liquid or gas phase or by NMR, but so far, we are unaware of any study that has used such approach to discover biomarkers of cereal intake. Metabolite features of special interest are then annotated/identified at a later stage in the analytical pipeline. Inherent to the wide coverage, untargeted approaches are well suited for exploratory biomarker studies, and this approach has been used to mine for dietary exposure biomarkers reflecting total or specific whole-grain intake as well as specific grain-based foods after controlled interventions with specific foods or reported food intakes [50–53]. Targeted and untargeted approaches are complementary to each other and could both be useful to discover and validate dietary biomarkers. A typical workflow involves identification of putative biomarker candidates via an untargeted profiling approach followed by validation of the biomarkers in targeted, quantitative analyses applied preferentially in other study cohorts. Recently, Zhu et al. [54] combined untargeted and targeted metabolomics approaches to discover biomarkers of whole-grain wheat intake in urine samples after intake of whole-grain wheat bread vs refined wheat bread in a kinetic study in 12 subjects. A panel of urinary markers consisting of seven alkylresorcinol metabolites and five benzoxazinoid derivatives as specific biomarkers, along with five phenolic acid derivatives were suggested to reflect whole-grain wheat intake. Panels of biomarkers of whole grain, refined grain, or fractions of specific grains appear promising but remain to be evaluated in larger studies.

### Biomarkers of whole grains, cereal fractions, and specific foods

To date, only a few specific biomarkers have been suggested for different whole grains, bran fractions, or foods thereof and currently no biomarkers have been suggested for refined grains (Table 1). We are not aware of any compounds that specifically reflect total whole-grain intake from all cereals but a few compounds that are exclusively found in specific whole grains or fractions of specific grains do exist. For example, avenanthramides are only found in oats among cereals and odd-numbered alkylresorcinols are present in wheat, rye, and barley, with specific homolog profiles for the different grains mentioned. The concentrations of these molecules or their metabolites in plasma and urine have been suggested and used as biomarkers of intake (Table 1). Moreover, specific benzoxazinoids and their metabolites in plasma and/or urine have already been shown to be specific to wheat and rye (Table 1). In some cases, molecules that discriminate high vs low whole grain or bran intake vs refined grain after controlled or reported intake have been discovered in plasma and/or urine as metabolites, but they appear not to be specific. For example, different cinnamic acids such as ferulic acid derivatives are abundant in plasma and urine after wheat and rye bran consumption (Table 1). These molecules are found in high contents, mainly bound to the dietary fiber complex primarily in the bran, but are liberated by microbiota in the large intestine and absorbed [81, 91, 92]. They are not unique to any particular grain, they will appear as plasma biomarkers that differentiate between high vs low consumers of whole grain or wheat bran and rye consumers. Since these molecules are present in various other foods and, therefore, not specific to whole-grain intake, they are of limited use as specific dietary biomarkers.

**Table 1:** Studies reporting candidate biomarkers for cereal food intake

Discriminating metabolites/candidate biomarkers	Dietary factor	Study design	Number of subjects	Analytical method	Sample type	Primary reference(s)
Biomarkers of total whole grain (WG) intake						
Total ARs	WG products RG products	Intervention study, crossover, randomized (6 weeks)	30	GC-MS	Plasma	[30]
ARs	WG cereal	Observational study	33	GC-MS	Plasma	[55]
ARs	WG products RG products	Intervention study, parallel, randomized (12 weeks)	50	LC-MS/MS	Plasma	[56]
ARs	WG diet RG diet	Intervention study, crossover, randomized (6 weeks)	33	LC-MS/MS	Plasma	[57]
AR	WG products WG rye WG bread	Observational study	522	GC-MS	Plasma	[58]
ARs Betaine	WG diet RG diet	Intervention study, crossover, randomized (2 weeks)	17	GC-MS/MS (ARs) LC-MS/MS (betaine)	Plasma	[59]
Total ARs (C17:0, C19:0, C21:0, C23:0, C25:0)	WG products Habitual diet	Intervention study, parallel, randomized (16 weeks)	316	GC-MS	Plasma	[31]
Total AR AR (C17:0-C25:0)	WG products	Observational study	360	GC-MS	Plasma	[60]
AR (C17:0, C19:0, C21:0, C23:0, C25:0)	WG products	Observational study	407	LC-MS	Plasma	[61]
AR (C17:0-C25:0)	WG products	Observational study	20	GC–MS	Adipose tissue	[62]
DHBA DHPPA DHBA+DHPPA	WG products Cereal fiber	Observational study	104	GC-MS	Urine	[63]
DHBA DHPPA Total metabolites	WG foods Cereal fiber	Observational study	66	GC-MS	Urine	[64]
DHBA DHPPA	WG products Fiber	Observational study	2833	HPLC	Plasma	[65]
DHPPA	WG products WG rye and wheat	Observational study	100	HPLC-CEAD	Urine	[66]
2-Aminophenol-Slf HPAA-GlcA HHPAA HMBOA-GlcA HBOA glycoside HPPA HMBOA DHPPA-GlcA 3,5-Dihydroxyphenylethanol-Slf DHPPTA-Slf Hydroxybenzoic acid-Slf Dihydroferulic acid-Slf Enterolactone-GlcA Pyrraline 3-Indolecarboxylic acid-GlcA Riboflavin *N*-α-Acetylcitrulline 2,8-Dihydroxyquinoline-GlcA	White bread WG bread	Observational study	155	HPLC-q-TOF-MS	Urine	[67]
5-Nonadecyl-1,3-benzenediol-GlcA (AR) 5-(16-Heneicosenyl)-1,3-benzenediol-GlcA	WG products, fatty fish and bilberries diet WG diet Refined wheat bread diet	Intervention study, parallel, randomized (12 weeks)	106	UHPLC-q-TOF-MS	Plasma	[53]
Proline Ornithine Arginine	Grain protein-based diet (wheat, bran, rice, and maize) Dairy protein-based diet (milk and milk products, yogurt and cheese) Meat protein-based diet (pork, beef, and chicken)	Intervention study, crossover, randomized (1 week)	100	LC-tripleQ-MS	Plasma	[68]
Biomarkers of whole-grain wheat and rye intake						
Total ARs	WG rye bread and WG wheat bread Gluten-free diet	Intervention study, parallel (2 weeks)	9	GC-MS	Plasma and erythrocyte membranes	[69]
Total ARs	WG rye and wheat Cereal fiber	Observational study	51	GC-MS	Plasma	[70]
Total ARs AR C17:0, C19:0, C21:0, C23:0, C25:0 Enterolactone	Rye bread Wheat bread	Intervention study, crossover, randomized (8 weeks)	39	GC-MS	Plasma	[71]
Total AR (C17:0, C19:0, C21:0, C23:0, C25:0) AR C17:0/C21:0 Enterolactone	WG wheat WG rye crisp bread	Intervention study, crossover, randomized (1 week)	15	GC-MS	Plasma (AR) erythrocyete membrane (AR) Lipoproteins (AR) Serum (enterolactone)	[69]
Total ARs (C17:0–C25:0) AR C17:0/C21:0	Nordic diet (rich in WG rye and wheat) Control diet	Intervention study, parallel, randomized (18–24 weeks)	158	GC-MS	Plasma	[32]
Total ARs AR C17:0/C21:0	WG wheat RG wheat	Intervention study, parallel, randomized (12 weeks)	72	LC-MS/MS	Plasma	[72]
DHBA DHPPA	Rye bread Bread fiber	Observational study	122	HPLC-CEAD	Urine Plasma	[73]
DHBA DHPPA	Rye bread with plant sterols Rye bread	Intervention study, parallel, randomized (4 weeks)	68	HPLC-CEAD	Plasma	[74]
DHBA DHPPA	Rye bread	Intervention study (postprandial trial)	15	HPLC-CEAD	Urine	[75]
DHBA DHPPA	Rye bread	Intervention study (postprandial trial)	15	HPLC-CEAD	Plasma	[76]
DHBA DHPPA	Rye	Observational study	60	HPLC-CEAD	Urine Plasma	[77]
DHBA DHPPA	WG rye and wheat	Observational study	52	GC-MS	Urine	[78]
DHBA DHBA glycine DHPPA DHPPTA	RG wheat bread, WG wheat bread	Intervention study (postprandial trial)	12	LC-MS HPLC-CEAD	Urine	[79]
HHPAA-GlcA HHPAA-Slf HPAA-Slf HBOA-GlcA Phenylacetylglutamine derivative Creatinine *N*-feruloylglycine-Slf	WG rye foods	Intervention study (8 weeks)	33	FIE-MS	Urine	[80]
DHFA Ferulic acid	WG wheat products Refined wheat products	Intervention study, parallel, randomized (8 weeks)	80	HPLC-MS/MS	Urine Serum Feces	[81]
3,5-Dihydroxyhydrocinamic acid sulfate Ascorbic acid 2-Aminophenol-Slf Nonanedioic acid DHPPA-GlcA Indolylacryloylglycine Enterolactone-GlcA DHPPA-Slf Ferulic acid-4-Slf 2,4-Dihydroxy-1,4-benzoxazin-3-one-Slf 3,5-Dihydroxyphenylethanol-Slf 1,3,4,5-Tetrahydroxycyclohexane-1-carboxylic acid Others non-identified metabolites	WG rye bread Refined wheat bread	Intervention study, crossover, randomized (4 weeks)	20	UPLC-q-TOF-MS	Urine	[50]
Biomarkers of specific fractions of foods of wheat and rye intake						
Total ARs AR (C17:0, C19:0, C21:0, C23:0, C25:0)	WG grains Cereal fiber Bran Total fiber Legume fiber	Observational study	165	GC-MS	Plasma	[82]
Total ARs AR C17:0/C21:0	Cereal fiber	Observational study	2845	GC-MS	Plasma	[83]
Total ARs AR C17:0/C21:0	Cereal fiber	Observational study	2744	GC-MS	Plasma	[84]
Total AR AR C17:0/C21:0	Rye WG/bran Refined wheat	Intervention study, crossover, randomized (6 weeks)	17	GC-MS	Plasma	[85]
AR C23:0 AR C25:0 AR C17:0/C21:0	Rye bran flakes	Intervention study (postprandial trial)	6	GC-MS	Plasma	[86]
ARs (plasma) DHBA (urine) DHPPA (urine)	Cereal fiber	Observational study	56	HPLC-CEAD	Urine Plasma	[87]
DHBA DHPPA	Cereal fiber	Observational study	56	HPLC-CEAD	Plasma	[88]
HPAA-Slf HHPAA-Slf	WG sourdough rye bread White bread with native unprocessed rye bran White bread with bioprocessed rye bran White bread	Intervention study, crossover, randomized (postprandial trial)	12	LC-q-TOF-MS	Plasma	[52]
2,6-dihydroxybenzoic acid 2-aminophenol-Slf	High fiber diet Low fiber diet	Intervention study, crossover, randomized (5 weeks)	25	UPLC-q-TOF-MS	Plasma	[89]
Ferulic acid	Rye bran bread Inert wheat bran	Intervention study, crossover, randomized (6 weeks)	18	HPLC	Urine	[90]
Ferulic acid Vanillic acid Sinapic acid 3,4-Dimethoxybenzoic acid Phenylpropionic acid 3-Hydroxyphenylpropionic acid	Whole wheat bread with bioprocessed bran Whole wheat bread with native bran	Intervention study, crossover, randomized (postprandial trial)	8	GCxGC-q-TOF-MS	Urine Plasma	[91]
Ferulic acid Sinapic acid	White wheat bread with bioprocessed rye bran White wheat bread with native rye bran	Intervention study, crossover, randomized (postprandial trial)	15	GC-MS	Urine	[92]
Indole-2-carboxylic acid Hydrocinnamic acid α-tocopherol Benzoic acid Cycloartenol Pantothenic acid Phenylacetic acid β-sitosterol	Heat-stabilized rice bran	Intervention study, parallel, randomized (4 weeks)	7	GC-MS	Feces	[93]
Biomarkers of quinoa						
Even-numbered AR	Quinoa	Intervention study	NS	LC-MS/MS GC-MS/MS	Plasma	[94]
Biomarkers dependent on gut microbiota						
Enterolactone	High-fiber bread Fiber Fruits and berries	Observational study	1099	TRFIA	Plasma	[95]
Enterolactone	WG products	Observational study	1889	TRFIA	Plasma	[95]
Enterolactone	High-fiber rye High-fiber wheat Low-fiber foods	Intervention study, crossover, randomized (4 weeks)	28	TRFIA	Plasma	[96]
Enterodiol	WG diet RG diet	Intervention study, crossover, randomized (12 weeks)	13	HPLC-CEAD)	Urine	[97]
Enterolactone Enterodiol	Quinoa flakes Corn flakes	Intervention study, parallel, randomized (4 weeks)	35	HPLC	Urine Serum	[98]
Enterodiol (serum) Enterolactone (serum & urine)	Flaxseed Rice	Intervention study, parallel (6 weeks)	27	HPLC	Urine Serum	[99]

*Abbreviations*: *AR* alkylresorcinols, *CEAD* coulometric electrode array detection, *DHBA* 3,5-dihydroxy-benzoic acid, *DHBA glycine* 2-(3,5-dihydroxybenzamido)acetic acid, *DHFA* dihydroferulic acid, *DHPPA* 3-(3,5-dihydroxyphenyl)-1-propanoic acid, *DHPPTA* 5-(3,5-dihydroxyphenyl)pentanoic acid, *FIE* flow infusion electrospray-ionization, *GC* gas chromatography, *GCxGC* two-dimensional GC, *GlcA* glucuronide, *HBOA* 2-Hydroxy-1,4-benzoxazin-3-one, *HHPAA* 2-hydroxy-*N*-(2-hydroxyphenyl) acetamide, *HPAA N*-(2-hydroxyphenyl) acetamide, *HPLC* high-performance liquid chromatography, *LC* liquid chromatography, *MS* mass spectrometry, *MS*/*MS* tandem mass spectrometry, *NS* not specified, *q-TOF* quadrupole time-of-flight, *RG* refined-grain, *Slf* sulfate, *TRFIA* time-resolved fluoroimmunoassay, *WG* whole-grain

#### Alkylresorcinols and their metabolites—biomarkers of whole-grain wheat and rye intake

Alkylresorcinols comprise a group of phenolic lipids that have been suggested and evaluated as specific biomarkers of wheat and rye intake [100]. They are also found in small amounts in barley [46]. Alkylresorcinols are located in the waxy cuticula in between the seed and fruit coats of wheat and rye and are therefore a specific biomarker of bran from these cereals in studies where other whole grains are not consumed [100]. However, bran per se is not often consumed but is usually consumed as whole grain. A large number of studies have been undertaken to evaluate alkylresorcinols as biomarkers after self-reported intake or controlled dietary interventions with different whole-grain wheat and rye products (Table 1). The results suggest that alkylresorcinols can be used as biomarkers of whole-grain wheat and rye, irrespective of food in what food matrix they are present in [19, 29, 32].

Also, alkylresorcinol metabolites in urine and plasma have been suggested to reflect the intake of whole-grain wheat and rye [101, 102]. Due to their unique presence in the outer parts of wheat and rye grains, alkylresorcinols (AR) are present not only in bran, but also in whole grain (due to the presence of bran), but only in minute amounts in refined flour [100]. Since alkylresorcinols are not degraded in food processing, they will appear in quantities related to the amount of specific whole grain and bran consumed.

The sum of dominant alkylresorcinol homologs with alkyl-chains in the range 17–25 carbon atoms in plasma, i.e., total plasma alkylresorcinol concentration, reflects total whole-grain wheat and rye intake in a dose-dependent manner [85]. The alkylresorcinol homolog profile is specific for common wheat, durum wheat, and rye, and the ratio of two specific alkylresorcinol homologs (C17:0/C21:0) can be used as a biomarker of the intake of whole-grain rye to whole-grain wheat intake, since the ratio is always 1.0 in whole-grain rye-based foods, 0.1 in common wheat and 0.01 in durum wheat [30, 31, 71, 85, 103, 104]. Plasma alkylresorcinols have a rather short apparent elimination half-life (4–6 h) and thus reflect medium- to long-term whole-grain wheat and rye intake in populations with stable and frequent intake [29, 105] but are less suitable in populations where intake is less frequent [82, 106].

Alkylresorcinols are metabolized by CYP450-dependent metabolism, which causes insertion of an OH-group at the end of the alkyl-chain, followed by subsequent oxidation into a COOH-group. This derivative then undergoes beta-oxidation, where the side-chain is degraded by stepwise removal of C_2_H_5_ per cycle, generating free and conjugated 1,3-dihydroxy-benzoic acid (DHBA) and 3-(1,3-dihydroxyphenyl)-1-propionic acid (DHPPA) as the main end products [102]. Urinary excretion of DHBA and DHPPA has been shown similar validity as plasma AR concentrations in free-living subjects with high and frequent intake [64, 87]. As expected, spot urine samples fluctuated more day to day and were somewhat less correlated with estimated intake compared with 24-h collections [63, 64, 66]. Some studies report that DHBA and DHPPA are also present in urine after intake of food compounds not derived from cereals [34]. This could have implications for the use of DHBA and DHPPA as biomarkers of whole-grain wheat and rye intake, particularly in populations with low intake. However, intakes have been correlated with levels of DHBA and DHPPA in urine from the US population where the intake of whole grain is small [63].

Recently, new alkylresorcinol metabolites (DHBA-glycine, DHPPTA, DHCA) were detected in urine from mice and/or humans and their half-lives were suggested to be longer than that of previously identified alkylresorcinol metabolites [50, 79, 101]. These biomarkers were evaluated in free-living Swedish men and women and results showed that DHPPTA and DHCA determined in single 24-h urine excretions had excellent reproducibility (ICC = 0.63 for both) and good relative validity (*r* = 0.40–0.65), and thus could be useful as long-term biomarkers of whole-grain wheat and rye intake [102]. However, 24-h urine collections are typically not available in large scale epidemiologic studies, but spot urine samples may be more readily available. It is therefore highly relevant to asses if these biomarkers analyzed in spot urine samples remain useful as biomarkers of wheat/rye whole-grain intake [63, 107].

#### Benzoxazinoids—potential biomarkers of whole-grain wheat and rye as well as for sourdough rye

Benzoxazinoids is another group of compounds that comprise specific derivatives almost exclusively found in wheat and rye grains. These compounds have been originally described in the context of the defense mechanism of certain plant species including rye, wheat, and maize [108]. Nowadays, they are studied as part of the dietary compound repertoire related in particular to whole-grain wheat and rye. Dihm et al. [109] conducted a detailed study where major benzoxazinoid compounds were quantified in various grain-based products namely commercial flours (whole-grain wheat flour, coarse-rye flour, fine-rye flour, refined-wheat flour, graham flour, quinoa flour, teff flour) and 20 commercial breads from Scandinavia, as well as 3 traditionally home-baked breads. The highest amount of benzoxazinoid compounds were found in flour from fine-rye flour (3.6 mg/g dry weight), whereas two Finnish rye breads had the highest amount among the bread products (2.3–3.3 mg/g dry weight). In all cases, the double-hexose conjugated 2,4-dihydroxy-1,4-benzoxazin-3-one (DIBOA) was the main benzoxazinoid metabolite.

The concentrations of specific benzoxazinoids and their metabolites in biofluids are largely affected by factors other than the whole-grain content, such as processing. For example, the double-hexose conjugated compounds abundant in flour are easily degraded during sourdough fermentation [110]. Interestingly, in the study by Dihm et al., [109] the level of double-hexose conjugated forms of benzoxazinoids was very high in two commercial Finnish breads, which further highlights the fact that the processing method can remarkably affect the chemical composition and dietary intake thereafter [108]. Few studies have investigated the concentration of benzoxazinoids in plasma and urine samples after whole-grain consumption (Table 1). Hanhineva et al. showed modest correlations between estimated whole-grain rye intake and benzoxazinoid in 24-h urine, but the levels were found to fluctuate extensively over a period of 2-3 months [51]. Other studies have shown increased plasma, urine, and tissue benzoxazinoid concentrations after intake of benzoxazinoid-rich foods from wheat and rye [50, 52]. A particular metabolite derived from benzoxazinoids via metabolism by the gut microbiota, and conjugation in the liver, is aminophenol sulfate. This compound has been reported in urine after intake of various bread products. Therefore, it appears that native benzoxazinoids present in the grains undergo massive conformational changes during technological processing a gut fermentation, as well as endogenous metabolism, and the kinetics as well as particular chemical conversions of the whole pathway are not yet known. Thus, more studies are needed to evaluate the feasibility of individual benzoxazinoids and their metabolites as biomarkers of WG intake.

Studies suggest that dietary benzoxazinoids are converted into phenylacetamides (2-hydroxy-*N*-(2-hydroxyphenyl)acetamide (HHPAA) and *N*-(2-hydroxyphenyl)acetamide (HPAA)) that are detectable in urine and plasma after consumption of a meal rich in whole grains [52]. Steffensen et al. investigated the concentration of different benzoxazinoids after intake of benzoxazinoid-rich foods from rye (flakes, porridge, and breads) in plasma, urine, and in prostate tissue in men with prostate cancer [111]. The overall finding was that benzoxazinoids increased in all matrices after high-benzoxazinoid-based rye foods, but different forms dominated in different matrices and varied between subjects. Beckmann et al. [80] used flow infusion electrospray mass spectrometry (MS) to profile metabolites in urine from participants who reported high intakes of rye flakes, rye pasta, or total whole-grain rye products, but they could not find any discriminative metabolites compared with subjects wash-out samples. However, they observed discrimination in urine samples from participants who reported high whole-grain sourdough rye bread consumption. They found that benzoxazinoid lactam 2-hydroxy-1,4-benzoxazin-3-one and hydroxylated phenyl acetamide derivatives were higher after sourdough rye bread consumption and that these molecules may be candidate biomarkers of such foods. However, as noted by Hanhineva et al. [52], bioprocessing such as baking that involves microbial metabolism (e.g., sourdough fermentation) has a central role in modulating the phytochemical content in whole-grain and bran-rich breads and it is likely that differences in the processes and inclusion of different starters etc. may cause variation in suggested biomarkers. Thus, biomarkers that may be valid for one type of product may not be universal to all.

Only very few studies have investigated whether benzoxazinoids or their metabolites could be used as biomarkers of whole-grain wheat and rye intake or for specific foods, such as sourdough-fermented rye bread. In one study, their levels in urine samples were well correlated with estimated whole-grain rye intake [53], but they were found to fluctuate considerably in urine samples taken 2−3 months in between, probably due to short half-lives. Further studies are needed to validate benzoxazinoids as biomarkers in both controlled feeding-trials and in observational studies. Correlations with estimated intakes as well as stability over time need to be estimated to assess their usefulness as biomarkers. Indeed, the involvement of the gut microbiota in benzoxazinoids metabolism necessitates further study to establish which microorganisms or groups of microorganisms may be involved. Recent observations that certain metabotypes (e.g., urolithin metabotypes) determined by gut microbiota metabolism of other polyphenols may not be stable over time but change with age, habitual dietary intake, obesity, disease state, etc. suggest that gut microbiota community structure and metabolic output are closely linked but not fixed for a given individual [112]. This also has implications for the validity of small phenolic acids as biomarkers of intake, if their production from benzoxazinoids for example, changes as gut microbiota composition changes. Similarly, since we still know little about how specific polyphenol-derived small phenolic acids impact human physiology, the significance of pliable metabotypes in terms of human health remains to be determined.

### Biomarkers of whole-grain oat intake

Until recently, there have been no biomarker candidates of whole-grain oat intake. Oats contains two classes of unique compounds: avenanthramides (AVAs) and steroidal saponins. AVAs are substituted *N*-cinnamoylanthranilic acids consisting of anthranilic acid and cinnamic acid moieties. To date, 25 AVAs which differ in the substitution patterns of two moieties have been identified in oats; some at very low concentrations [113]. The most common avenanthramides are AVA-A (2p), AVA-B (2f), and AVA-C (2c) and differ only by a single moiety on the hydroxycinnamic acid ring. Several studies have evaluated the uptake of avenanthramides in humans and these studies found that different avenanthramides show different, but consistently low, bioavailability in humans. Chen et al. [114] reported that serum levels of AVA 2p, 2f, and 2c reach a peak 2 h after consumption of an AVA-enriched mixture (AEM) with a gradual return to base-line within 10 h. Recently, Zhang et al. [115] showed for the first time that AVAs were bioavailable in humans, after consumption of cookies based on regular oat flour. Previous studies had used AVA-enriched fractions or extracts [115]. AVA-B has the slowest elimination rate and the longest half-life compared to AVA-A and AVA-C. The half-lives were in the range 2–5 hours [116]. AVAs like other phenolic compounds are extensively metabolized. Walsh et al. [116] fed 12 subjects with muffins with oat bran enriched in AVA and investigated potential metabolites of AVAs in plasma. They identified two methylated AVAs but did not detect any sulfate- or glucuronide conjugates.

Schär et al. [117] studied the excretion of phenolic acids and avenanthramides in urine samples among seven subjects after consumption of 60 g of oat bran compared to a control diet low in phenolic compounds. In total, 30 compounds were higher in urine up to 8 h after oat bran consumption. Vanillic acid, 4- and 3-hydroxyhippuric acids, and sulfate-conjugates of benzoic and ferulic acids were the major compounds excreted. Sang et al. [49] investigated whether AVAs and their metabolites could be used as exposure markers for whole-grain oat intake. They identified a reduction of the double bond in the cinnamic acid unit and cleavage of the amide bond as the major metabolic pathways of AVAs, that the double bond reduced metabolites (DH-AVAs) were derived from gut microbiota. Excretion in urine suggested that the DH-AVAs had longer half-life and that the combination of AVAs and DH-AVAs may better reflect long-term intake and may jointly be used as biomarkers of whole-grain oat intake. However, not all participants produced DH-AVAs which suggest that microbiota is an important determinant that may need to be taken into account. However, as with other cereals, we still know little about which bacteria or groups of bacteria are involved, and how their production of these metabolites changes with age, sex, health status, or xenobiotic (e.g., drug) exposure.

Oat contains two unique steroid glycosides, avenacoside-A (AVE-A) and AVE-B [113, 118]. AVE-A and -B are present in high concentrations in oat bran products with a total content of AVE-A and -B that varies from 304 to 443.0 mg/kg [118]. To our knowledge, only one study has been reported where the metabolic fate of AVEs has been investigated [48]. Wang et al. analyzed AVE-A and B in urine from 12 individuals during 48 h after a single dose of oat bran and they also assessed the potential impact of the human gut microbiota. The aim was to evaluate the potential of these molecules as putative biomarkers of whole-grain oat intake. The concentrations of AVE-A and -B increased rapidly after oat bran intake. The average apparent half-lives were 4.5 h and 6.2 for AVE-A and -B, respectively. Deglycosylation was identified as the major metabolic path for AVE-A and -B metabolism in experiments where pure AVE-A and -B were incubated with human fecal samples. Both human and mice gut microbiota metabolized AVE-A and -B in a similar way and 3 metabolites of AVE-A and 5 metabolites of AVE-B were detected from both man and mice [48]. The total 24-h urinary recovery of AVE-A and -B was <5% of ingested dose. The influence of gut microbiota on AVE-A and -B may affect their validity as biomarkers, but further studies need to be conducted to evaluate this.

Both avenanthramides, avenacosides, and their metabolites may have potential as short-to-medium-term biomarkers of oat intake, since they are not found in other commonly consumed foods. However, they show low bioavailability and rapid metabolism which is partly dependent on gut microbiota. This probably affects their potential as biomarkers of oat intake, but validation studies are needed to confirm biomarker status [34].

### Biomarkers of quinoa intake

Quinoa is a pseudocereal typically grown in the Andes, but consumption is expanding especially in Europe mainly because of its nutritional profile and its use as a gluten-free alternative to cereal grains. Moreover, quinoa is not short in lysine, which increases the bioavailability of its amino acids, and could increase the nutritional value of gluten-free diets [119, 120]. Recently, Ross et al. discovered and profiled alkylresorcinols in 17 commercial samples of quinoa [47]. Interestingly, the authors found a surprising AR profile in quinoa samples, with about 30 alkylresorcinol derivatives including odd-, even-, and branched-chain alkylresorcinols as well as methyl-alkylresorcinols. The total AR contents in the quinoa samples were 58 ± 16 μg/g (AR), 182 ± 52 μg/g (branched-chain alkylresorcinols) and 136 ± 40 μg/g (methyl-alkylresorcinols) [47]. These values were much lower than those reported in rye and wheat but in a similar magnitude as those quantified in barley [121]. Some of the alkylresorcinol homologs in quinoa are also present in other cereal species but the unique alkylresorcinol homolog composition profile with even-numbered alkylresorcinol homologs in quinoa allows its discrimination from those alkylresorcinol derived from wheat, rye, and barley [122]. It should be noted that some of the compounds were identified for the first time in nature. Among the even-numbered alkylresorcinol homologs, C18:0, C20:0, C22:0, and C24:0 are commonly present in quinoa but not in other cereals [47]. In order to evaluate whether even-numbered alkylresorcinols in plasma could be used as biomarkers of quinoa intake, Ross et al. [47] applied a liquid chromatography tandem mass spectrometry (LC-MS/MS) method to identify and quantify the even-chained alkylresorcinols in plasma from a volunteer 12 h after consuming 100 g (uncooked weight) of white quinoa. The authors showed that the concentrations of these metabolites were higher in plasma after quinoa consumption. Alkylresorcinol C22:0 had previously been described in volunteers following a crossover intervention with a gluten-free diet, possibly due to quinoa intake [122]. In conclusion, since quinoa appears to be an exclusive source of the even-chain alkylresorcinols and because these compounds have been detected and quantified in plasma samples following quinoa intake, it is feasible to propose these compounds as biomarkers of quinoa intake. However, validation studies to assess half-life, dose response, reproducibility, and validity under controlled intake and under free-living condition are needed.

### Biomarkers of rice intake

Very few studies have been reported where putative biomarkers of rice intake were explored. Guertin et al. [123], analyzed baseline serum samples from 502 participants in the Prostate, Lung, Colorectal, and Ovarian (PLCO) Cancer Screening Trial with LC-MS/MS and gas chromatography mass spectrometry (GC-MS). They detected 412 known metabolites and correlated these to different food intakes, reported by FFQ, including rice intake. Among investigated metabolites, only docosahexaenoic acid (DHA) correlated significantly with rice intake, and this was likely due to confounding by fish intake. Li et al. [124] analyzed the plasma metabolome in 38 children after 4-week intake of rice bran in one arm of a study to investigate its impact on cholesterol concentrations in plasma. The authors analyzed 854 metabolites in plasma and about 300 were also found in the rice bran food metabolome. Rice bran metabolites detected with high relative abundance in plasma included methionine sulfone, alpha-hydroxycaproate, linoleoyllinolenoyl-glycerol, palmitoyl-linolenoylglycerol, pyridoxal, 2-hydroxyhippurate, salicylate, gamma-glutamylglutamate, gamma-glutamylthreonine, hypoxanthine, and dihydroorotate. However, it is unclear to what extent these metabolites, separately or in combination, would specifically reflect rice bran intake.

In another study [125], the same group applied GC-MS-based metabolomics on stool samples from 19 colorectal cancer survivors who were fed heated rice bran or control for a period of 4 weeks. They found 39 metabolites that were higher after rice bran intake compared with baseline and which, at the same time, overlapped with the rice bran metabolome. These metabolites included lipid compounds, tryptophan metabolites, flavonoids, and B-vitamins, among other molecules. Although the authors suggest that rice bran-derived phytochemicals in plasma and stool samples may be used as biomarkers of rice bran intake, most metabolites are likely too unspecific to be used as specific biomarkers of rice bran intake. Further studies are needed to find biomarkers or biomarker panels that are specific to rice intake.

### Biomarkers of refined grains

To our knowledge, there are currently no suggested biomarkers of refined grain intake. This may be due to the fact that most bioactive compounds that could be putative biomarkers are typically located in the outer parts of the grains, and not in the starchy endosperm. The starchy endosperm in cereals contains low amounts of phytochemicals compared with the bran and germ [126]. Moreover, most studies that have been undertaken to find biomarkers of whole grains have used refined grains as a control and it is difficult to use an appropriate control for refined grains per se.

### Critical factors that affect biomarker validity and reproducibility—key features of biomarkers

Biomarker discovery needs to be followed by validation. Several criteria need to be fulfilled before a biomarker can be considered valid, and a framework for the validation of dietary biomarkers have been established [37]. Early validation may include assessment of how specific a biomarker is for a specific food, its pharmacokinetics, dose-response, and its non-dietary determinants. Validity and reproducibility are two features that to a great extent determine the usefulness of a biomarker. Validity is the lack of systematic measurement error when comparing the actual observation with that obtained using a reference method [127]. The correlation between a biomarker measurement and the true intake of the exposure of interest reflects the validity of the biomarker, but since true dietary exposure cannot be estimated without measurement error, the correlation only reflects the upper limit of the validity [128]. However, it should be kept in mind that validity often is study-specific, and therefore it is important to estimate the validity under different conditions in different populations. The reproducibility of a biomarker describes the correlation between samplings within the same individual on independent occasions [38]. The biomarker reproducibility is largely determined by the stability of the individual’s intake of the food/nutrient of interest and the elimination half-life of the biomarker. A short half-life can be compensated for by stable and continuous intake [129]. An ideal biomarker should be both valid and reproducible, i.e., plausible and robust and provide an accurate ranking of the intake. A large number of factors affect the accuracy of a biomarker and it is therefore important to evaluate the biomarker before it is used, in order to estimate its reproducibility and validity and identify the factors that affect these parameters. Most food biomarkers fall into the category of concentration biomarkers and the accuracy of such biomarkers are highly variable and dependent on differences in bioavailability of the biomarker substance within and between subjects, differences in metabolism, interactions with other dietary components, differences in distribution volume across subjects as well as the potential impact of gut microbiota on biomarker compounds [130]. It is therefore important to evaluate these aspects of each biomarker before using it as an accurate measure of the intake.

### Validation and application of cereal intake biomarkers

Only very few of the suggested biomarkers of cereal intake have been through rigorous validation. All putative biomarkers covered in this review fall into the category of food intake biomarkers and can be classified as concentration biomarkers [36, 38]. This means that specific intakes are correlated with the concentrations of the biomarkers in the biological matrix investigated and that the biomarkers are affected by inter-personal variation in bioavailability, absorption, metabolism, distribution, and elimination. Moreover, gut microbiota may affect biomarker concentration. Variation inherent to non-dietary factors may distort the intake-biomarker relationship and it is important to establish the impact of the different non-dietary determinants as part of the validation process before using the biomarker [60]. For most of the putative biomarkers of cereal intake, this variation remains to be determined. In fact, most of the suggested biomarkers have not been validated at all or only evaluated with regard to some of the criteria [37]. Alkylresorcinols and their metabolites are rare examples of food biomarkers that have been extensively validated as biomarkers of whole-grain wheat and rye intake in various matrixes (alkylresorcinols: plasma, erythrocytes, adipose tissue biopsies; metabolites: plasma and urine) (Table 1). Due to a short-half life (about 5 h), they mainly reflect short-term intake, unless the whole-grain wheat and/or rye intake is consumed consistently (> 2 times per day). Under such conditions, the concentration is stable in fasting plasma samples. Alkylresorcinol concentrations in plasma and adipose tissues as well as their metabolites in plasma and urine samples are well correlated with estimated whole-grain wheat and rye intakes with correlations in the magnitude 0.3–0.55, depending on the used measure of dietary intake [31, 60, 63, 64, 131]. Alkylresorcinols have been found to be a useful complement to traditional dietary assessment methods in several endpoint studies as well as measures as compliance in dietary interventions [29, 31, 82, 83]. But it remains to be tested whether they can be combined with other biomarkers to further improve their specificity and sensitivity. Moreover, alkylresorcinols in fecal samples have not yet been evaluated as biomarkers. To our knowledge, other biomarker candidates of whole grains, refined grain or fractions of different cereals have not yet been applied as such in endpoint studies. For candidate biomarkers of other grains, more fundamental validation is needed.

## Conclusion

Several biomarker candidates for whole-grain wheat, rye, and oats have been discovered as well as biomarker candidates of fermented rye bread. These biomarker molecules are uniquely found in biological samples from humans after consumption and can be traced down to actual compounds in the food. However, their validity and reliability, which will affect their usefulness as biomarker candidates in epidemiological studies, typically remains to be evaluated under controlled and free-living conditions in humans. Moreover, factors that explain within- and between-person variability in putative biomarkers need to be studied in order to understand their potential and limitations as specific food intake biomarkers. Collection of comprehensive data on lifestyle, health parameters, and OMICs-data including gut microbiota will facilitate the dissection of sources of inter-personal variation and improve the understanding of what factors contribute to inter-individual variation in the ADME of dietary biomarkers.

Metabolomics has enabled simultaneous (semi)quantitation of several biomarkers at the same time in a large number of samples. This allows multi-biomarker signatures to be used as biomarkers rather than single molecules. This approach may have a larger potential to improve specificity and should be further evaluated for different grain intakes. Future studies should evaluate the most suitable matrix (plasma, erythrocytes, urine, hair, nails, or adipose tissue) for determination of specific biomarkers that reflect long-term intake, which is of highest relevance in most diet and health studies.

## Data Availability

This is a review article and does not include original data on humans. Therefore, no original data has been deposited or could be made available. All data have been extracted from published articles referenced in this review.

## Abbreviations

AR: Alkylresorcinols
CEAD: Coulometric electrode array detection
DHBA glycine: 2-(3,5-dihydroxybenzamido)acetic acid
DHBA: 3,5-dihydroxy-benzoic acid
DHFA: Dihydroferulic acid
DHPPA: 3-(3,5-dihydroxyphenyl)-1-propanoic acid
DHPPTA: 5-(3,5-dihydroxyphenyl)pentanoic acid
FIE: Flow infusion electrospray-ionization
GC: Gas chromatography
GCxGC: Two-dimensional GC
GlcA: Glucuronide
HBOA: 2-Hydroxy-1,4-benzoxazin-3-one
HHPAA: 2-hydroxy-*N*-(2-hydroxyphenyl) acetamide
HPAA: *N*-(2-hydroxyphenyl) acetamide
HPLC: High-performance liquid chromatography
LC: Liquid chromatography
MS: Mass spectrometry
MS/MS: Tandem mass spectrometry
NS: Not specified
q-TOF: Quadrupole time-of-flight
RG: Refined-grain
Slf: Sulfate
TRFIA: Time-resolved fluoroimmunoassay
WG: Whole-grain
